# A “biphasic glycosyltransferase high-throughput screen” identifies novel anthraquinone glycosides in the diversification of phenolic natural products

**DOI:** 10.1016/j.jbc.2023.102931

**Published:** 2023-01-20

**Authors:** F. Ifthiha Mohideen, David H. Kwan

**Affiliations:** 1 Department of Biology, Centre for Applied Synthetic Biology, and Centre for Structural and Functional Genomics, Concordia University, Montreal, Quebec, Canada; 2 Department of Chemistry and Biochemistry, Concordia University, Montreal, Quebec, Canada; 3 PROTEO, Quebec Network for Research on Protein Function, Quebec City, Quebec, Canada

**Keywords:** combinatorial enzymatic synthesis, glycodiversification, glycosylation, glycosyltransferases, high-throughput screening, natural products, GT, glycosyltransferase, LC-MS/MS, liquid chromatography–tandem mass spectrometry, MS, mass spectrometry, UGT, UDP-dependent glycosyltransferase

## Abstract

The sugar moieties of many glycosylated small molecule natural products are essential for their biological activity. Glycosyltransferases (GTs) are enzymes responsible for installing these sugar moieties on a variety of biomolecules. Many GTs active on natural products are inherently substrate promiscuous and thus serve as useful tools in manipulating natural product glycosylation to generate new combinations of sugar units (glycones) and scaffold molecules (aglycones) in a process called glycodiversification. It is important to have an effective screening tool to detect the activity of promiscuous enzymes and their resulting glycoside products. Toward this aim, we developed a strategy for screening natural product GTs in a high-throughput fashion enabled by rapid isolation and detection of chromophoric or fluorescent glycosylated natural products. This involves a solvent extraction step to isolate the resulting polar glycoside product from the unreacted aglycone acceptor substrate and the detection of the formed glycoside by the innate absorbance or fluorescence of the aglycone moiety. Using our approach, we screened a collection of natural product GTs against a panel of precursors to therapeutically important molecules. Three GTs showed previously unreported promiscuity toward anthraquinones resulting in novel ε-rhodomycinone glycosides. Considering the pharmaceutical value of clinically used anthraquinone glycosides that are biosynthesized from an ε-rhodomycinone precursor, and the significance that the sugar moiety has on the biological activity of these drugs, our results are of particular importance toward the glycodiversification of therapeutics in this class. The GTs identified and the novel compounds they produce show promise toward new biocatalytic tools and therapeutics.

The diversity and the abundance of glycans in nature underscores their importance in biology (1, 2). Many therapeutically valuable compounds are decorated with sugars, highlighting the function that GTs play in their biosynthesis. Several natural product GTs are inherently promiscuous to substrates and thus can be challenged by non-native donor and acceptor molecules. This makes them valuable tools toward enhancing or altering the bioactivity of natural products by changing their glycosylation, a process otherwise known as glycodiversification (3, 4, 5, 6). The success of glycodiversification greatly depends on effective screening methods to identify promiscuous enzymes. However, screening for GT activity is difficult since the formation of glycosidic bonds does not produce a conveniently measurable signal like a change in absorbance or fluorescence (7).

Conventionally, the catalytic activity of GTs is assayed through high-performance liquid chromatography (HPLC) and/or analysis by mass spectrometry (MS). However, the throughput of this approach is limited by the long operation time of HPLC instrumentation. Consequently, researchers have come up with different screening strategies that use the release of the nucleoside diphosphate (NDP) or nucleoside monophosphate (NMP) by-product of Leloir GT–catalyzed reactions (6, 8, 9, 10, 11, 12). In an early study, Palcic and colleagues report a coupled spectrophotometric assay, in which the releasing NDP is coupled through two enzymes, pyruvate kinase and lactate dehydrogenase toward the oxidation of NADH, which is monitored at 340 nm (8) and this is widely used by other research groups (13, 14). In line with NDP/NMP by-product release, Promega Corporation has designed a general assay that measures the enzyme activity by adding the NDP/NMP-Glo reagent (UDP-Glo, GDP-Glo, and UMP/CMP-Glo). This results in ATP synthesis and is coupled with a luciferase reaction to produce light (11, 12). Apart from the coupled enzymatic assays, the detection of NDP by-product has also been assayed using fluorescent sensors that showed selective binding to the releasing NDP over NDP-sugar donor substrates (9, 10). Although these NDP/NMP-based GT screening tools are attractive in the context of their throughput and sensitivity, a few limitations include the fact that the signal measured is not directly linked to the formation of a glycosidic bond to the acceptor substrate along with the potential interference from sugar donor hydrolysis and complications in performing this assay with crude lysates as the presence of contaminating NDPs/NMPs would result in false positives (6).

We have addressed the shortcomings of these existing assays by developing a high-throughput screening strategy based on the changes in physical properties of natural product aglycones upon glycosylation. The aglycone moieties of many classes of small molecule natural product glycosides are very often lipophilic with a phenolic chemical structure. This makes them poorly soluble in polar solvents without the sugar moiety and often renders them inherently chromogenic or fluorescent. The polarity of these optically active small molecules significantly increases upon glycosylation (15). Our high-throughput assay uses a simple liquid–liquid extraction to separate the polar glycoside product of the GT-catalyzed reaction from the unreacted aglycone acceptor, allowing us to detect the product by the inherent absorbance or fluorescence of the aglycone moiety (Fig. 1). We optimized this biphasic glycosyltransferase high-throughput (BiG HiT) assay to screen the substrate scope of a variety of UDP-dependent GTs (UGTs) derived from several plant species.

**Figure 1:**
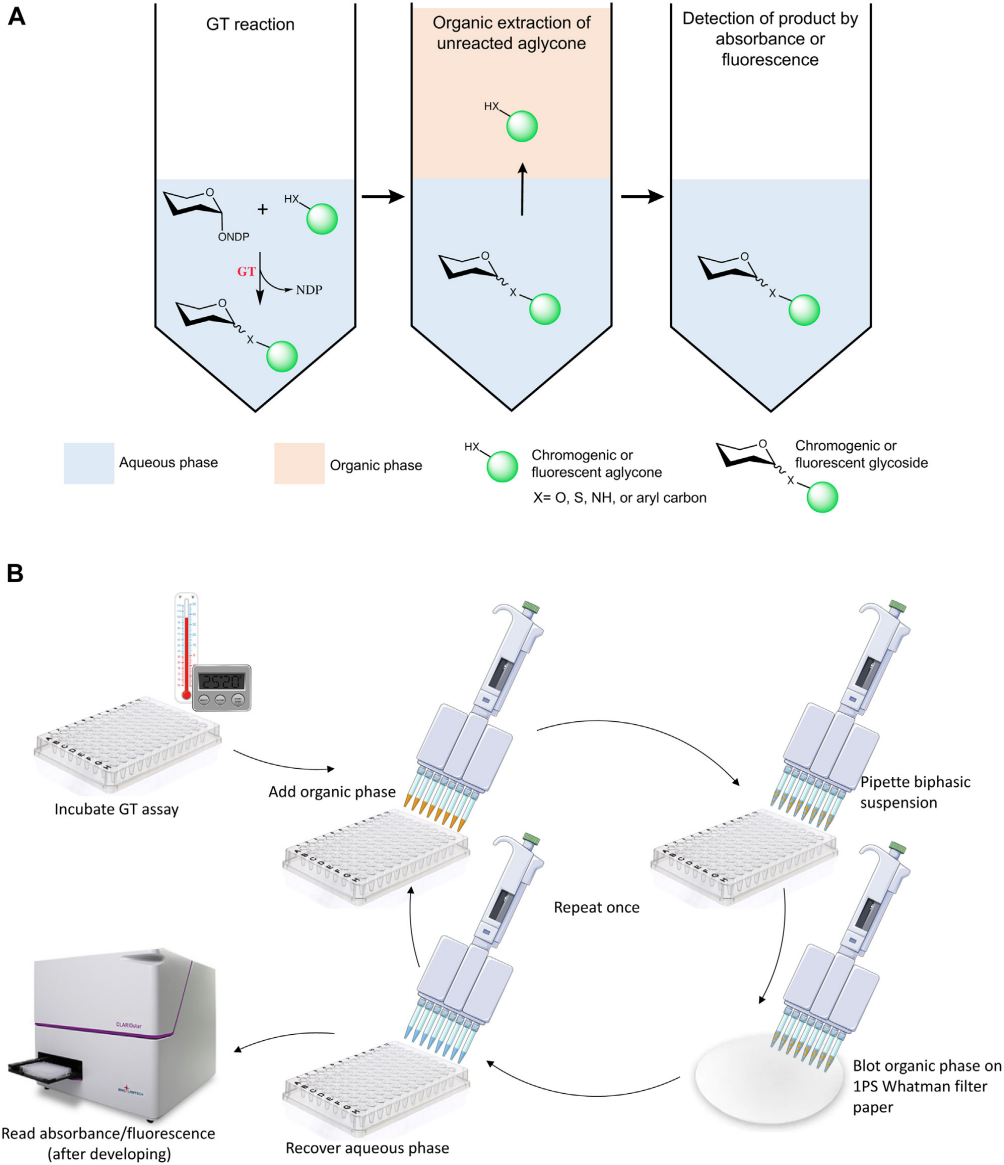
**Steps for performing the biphasic glycosyltransferase high-throughput (BiG HiT) assay.***A*, a schematic representation of the BiG HiT assay. A catalytically active glycosyltransferase (GT) transfers a sugar moiety from a nucleotide activated donor to a nonpolar naturally chromogenic or fluorescent acceptor and results in a chromogenic or fluorescent polar product (O-, C-, N-or S- linked). The unreacted aglycone is separated from the product by performing an organic solvent extraction. Subsequently, the product in the aqueous phase is detected by measuring the absorbance or fluorescence. *B*, a detailed representation of the steps of BiG HiT assay.

## Results

### Development and validation of the BiG HiT assay

**Figure 2:**
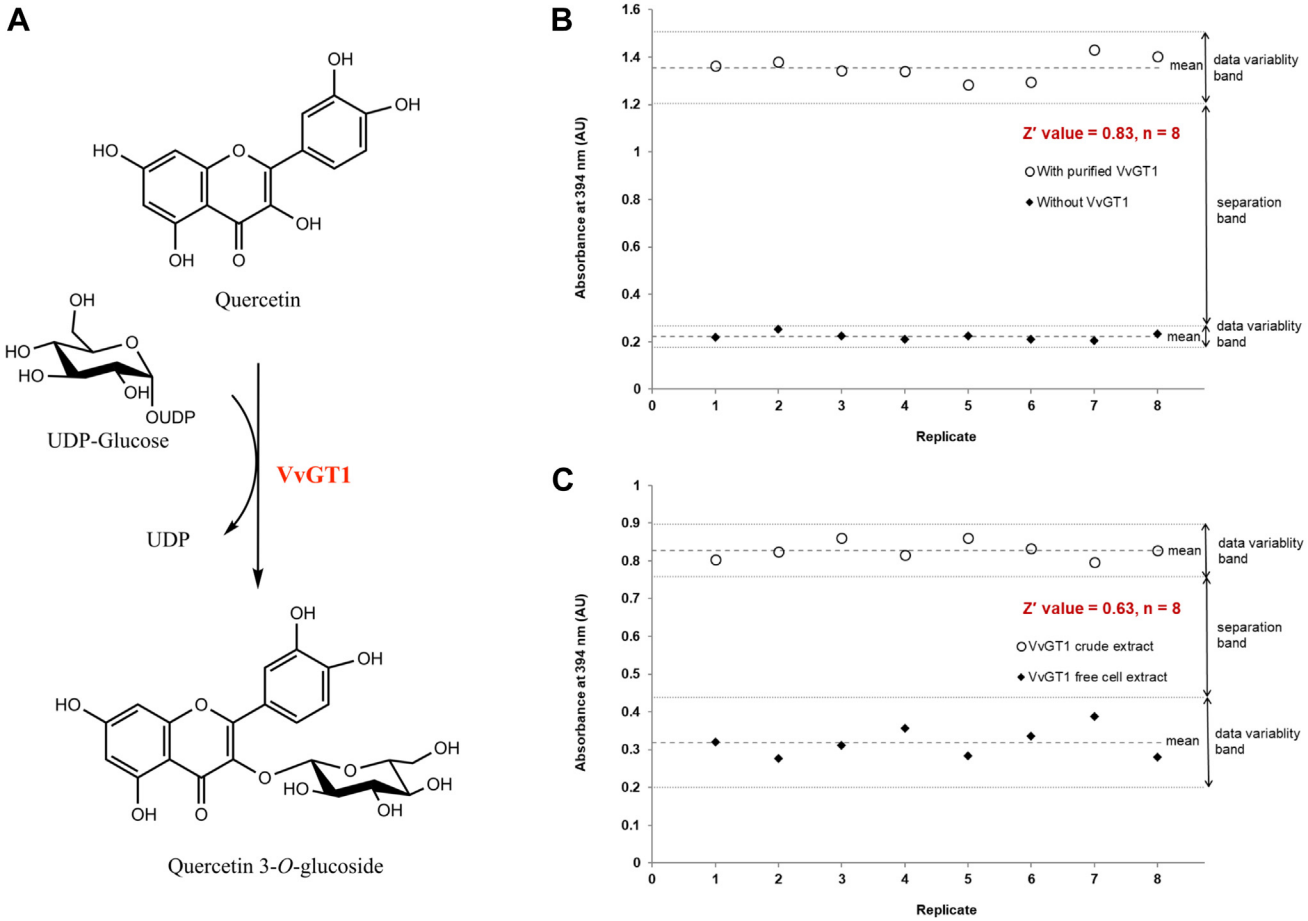
**Assay development and validation for high-throughput application.***A*, VvGT1-catalyzed glycosylation using UDP-glucose and quercetin. Statistical validation of the BiG HiT assay using *B*, VvGT1 purified enzyme (0.25 mg/ml) and *C*, crude extract. After solvent extraction, glycosylated quercetin 3-*O*-β-glucoside in aqueous phase was detected at 394 nm. Z′ was calculated from positive and negative controls. n = 8.

As a proof of concept, we used a promiscuous plant UGT, UDP-glucose:flavonoid 3-*O*-glycosyltransferase (VvGT1) from *Vitis vinifera* (red grape) (16, 17) to test the BiG HiT assay. Despite cyanidin being its natural acceptor, *in vitro* studies have shown VvGT1 can use other flavonoid acceptors such as quercetin (16). VvGT1 transfers the glucose moiety from the nucleotide activated sugar donor, UDP-glucose to the nonpolar aglycone acceptor, quercetin. This enzymatic reaction produces the yellow polar compound quercetin 3-*O*-β-glucoside (Fig. 2*A*).

We validated our screening strategy using purified VvGT1 protein (0.25 mg/ml). The reactions (eight replicates) were carried out in Tris buffer, pH 7.8 (6.7 mM) using quercetin (1 mM) as the acceptor and UDP-glucose (2 mM) as the sugar donor. To control for background from nonenzymatic reactions, an assay control buffer (50 mM Tris pH 7.5, 150 mM NaCl) without VvGT1 enzyme was used. Glycosylation reactions were performed for 2 h at 30 °C and terminated by heating at 90 °C for 3 min. Subsequently, the unreacted, less polar quercetin acceptor was removed from the solution containing the polar quercetin 3-*O*-β-glucoside product by an organic solvent extraction using ethyl acetate: chloroform (3:1 v/v). Solvent extraction was facilitated by the use of silicone-impregnated filter paper that is impermeable to the aqueous phase but permeable to the organic phase. The quercetin aglycone partitioned to the organic phase, which was absorbed by the silicone-impregnated filter paper while the aqueous phase containing the glycosylated product remained as a droplet. The separated polar quercetin 3-*O*-β-glucoside product was detected by measuring the absorbance of the aqueous phase at 394 nm after treatment with 2-aminoethyl diphenylborinate to enhance the absorbance signal of the flavonoid glycoside (18).

The Z-factor (or Z′; not to be confused with Z-score) is a statistical parameter that is used to measure the quality of high-throughput assays. This is calculated based on the variations between groups of positive and negative controls, and the value ranges from <0 to 1, where a value above 0.5 indicates an excellent assay (with the mean of positive and negative controls separated by >12 standard deviations) (19). We evaluated the robustness of our BiG HiT assay by calculating the Z-factor. As illustrated by Figure 2*B*, the calculated Z-factor value for the purified VvGT1 enzyme was 0.83. The compatibility with crude enzyme extracts would be necessary to use our assay for enzyme engineering strategies such as directed evolution. Thus, we tested the BiG HiT assay with crude extract of VvGT1 from *Escherichia coli* expression cultures (and crude extract from *E. coli* carrying empty vector as the negative control). This resulted in a Z-factor value of 0.63 (Fig. 2*C*).

### High-throughput combinatorial screening of the UGT collection with flavonol/anthraquinone acceptors and UDP-sugar donors

**Figure 3:**
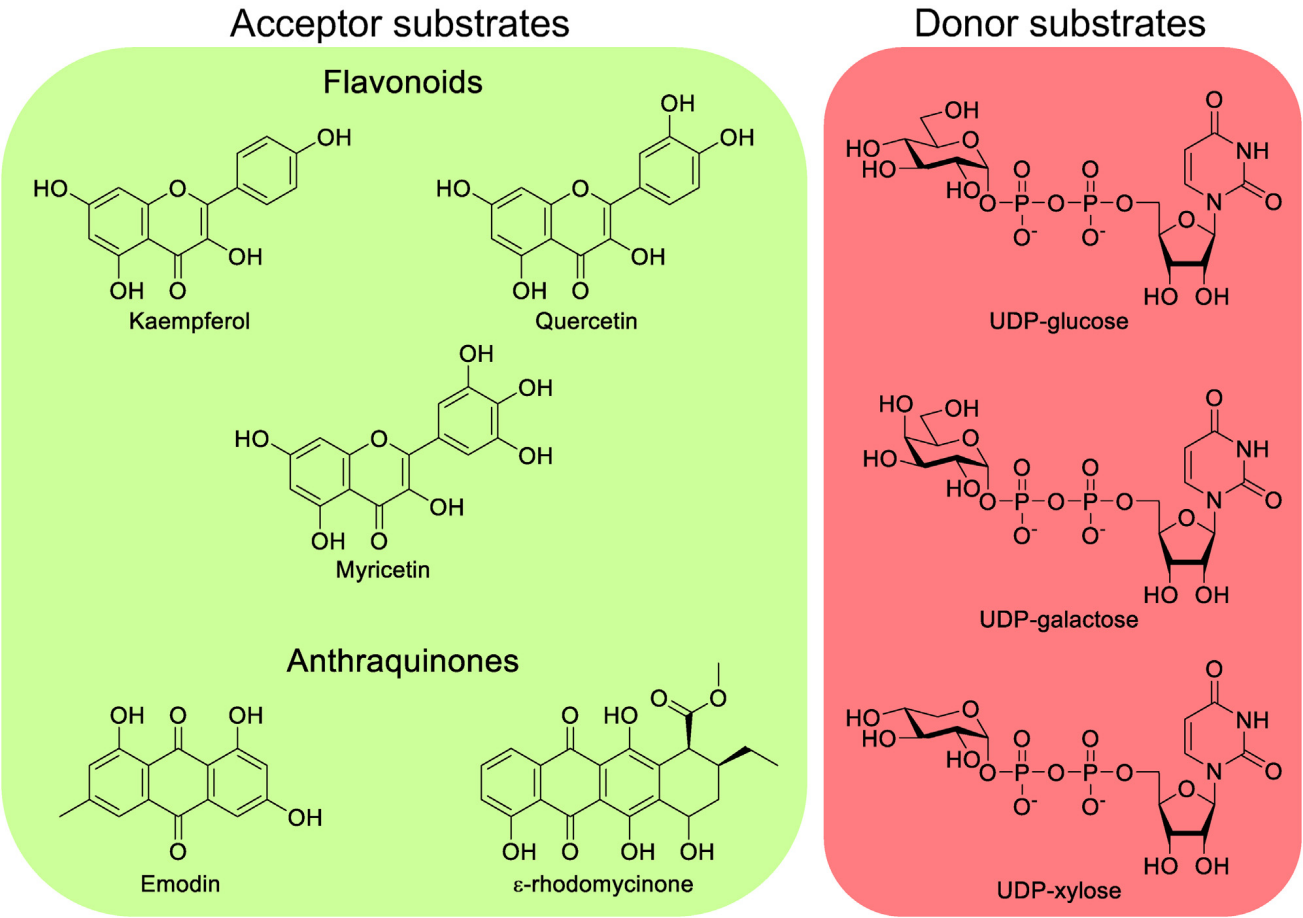
**Panel of substrates used in the BiG HiT assay including flavonoids (kaempferol, quercetin, and myricetin) and anthraquinones (emodin and ε-rhodomycinone) as acceptor substrates and UDP-glucose, UDP-galactose, and UDP-xylose as donor substrates**.

Subsequently, we screened a collection of recombinantly expressed and purified plant UGTs (Fig. S1) against a panel of natural product precursors to therapeutically important small molecule glycosides. Six substrate-flexible plant UGTs: VvGT1 from *V. vinifera*, UGT71G1 and UGT78G1 from *Medicago truncatula*, UGT78K6 from *Clitoria ternatea*, UGT708A6 from *Zea mays*, and UGT72B1 from *Arabidopsis thaliana* were selected as our enzyme collection. In nature, these UGTs exhibit different roles ranging from pigment production to plant defense mechanism and detoxification of environmental pollutants through glycosylation (16, 20, 21). As our substrate panel, three flavonoids that belong to the flavonol subcategory (kaempferol, quercetin, and myricetin) and two anthraquinones (emodin and ε-rhodomycinone) were used as acceptors and UDP-glucose, UDP-galactose, and UDP-xylose were the sugar donors (Fig. 3).

The conditions of the high-throughput combinatorial screen were the same as the assay validation, except two sets of negative controls without either UGTs or sugar donors were included. Furthermore, the unreacted nonpolar acceptor was removed from the polar glycoside using ethyl acetate:chloroform (3:1 v/v) or (1:3 v/v) for flavonol or anthraquinone, respectively, and instead of measuring absorbance of the aqueous phases at a fixed wavelength, we measured over selected ranges of wavelengths, which allowed us to detect the phenolic glycosides irrespective of their specific absorbance maxima. Indeed, testing this approach with quercetin 3-*O*-β-glucoside, we verified a linear relationship not only between concentration and absorbance at a specific wavelength (after adding 2-aminoethyl diphenylborinate) but also between concentration and the area under the absorbance curve at varied wavelengths (Fig. S2, *A* and *B*). This was also true for fluorescence measurements after development of the signal with 2-aminoethyl diphenylborinate (Fig. S2, *C* and *D*), which opens up the possibility of using fluorescence to perform highly sensitive readings for some phenolic compounds. Since we found that absorbance measurements were robust and rapid and required scanning of only one wavelength range as opposed to two for fluorescence (excitation and emission), we moved forward to do screening with absorbance measured over a range of wavelengths.

**Figure 4:**
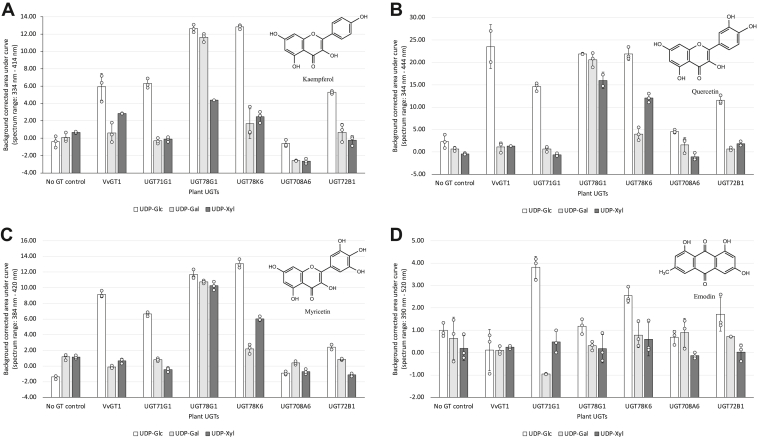
**Screening combinatorial glycosylation of phenolic natural products using the BiG HiT strategy.** Assay results for *in vitro* glycosylation of *A* kaempferol, *B* quercetin, *C* myricetin, and *D* emodin acceptors with different donor substrates by enzymes in the recombinant UGT collection. Screening was performed using the BiG HiT assay with a minimum of two replicates, and error bars represent one standard deviation. UGT, UDP-dependent glycosyltransferase.

Figure 4 summarizes the results of the BiG HiT combinatorial screen, which included hits from assays with all three flavonoids (kaempferol, quercetin, and myricetin) as well as those with the anthraquinone emodin, although none of the assays with the anthraquinone ε-rhodomycinone gave signal above background (not shown). If UGT enzymes were active in glycosylating the given acceptors with donors, we observed an increase in absorbance signal compared with the no-enzyme and no-sugar donor controls. The absorbance signal of reactions and no-GT controls were blank corrected by subtracting the respective no-donor controls. Any signal that was greater than the respective no-GT control was further analyzed by HPLC-MS/MS to validate hits and resolve ambiguous results (Fig. S3).

### Promiscuous substrate specificity of the UGT collection

When we screened the glycosylation activity of our UGT collection using the combinatorial BiG HiT assay, all six UGTs used UDP-glucose to glycosylate multiple acceptor molecules from the panel tested, as illustrated by Figure 4. We observed that a few enzymes use UDP-galactose in glycosylating at least two of the tested flavonol acceptors (Fig. 4, *A–C*). Interestingly, UGT78K6 showed previously unreported substrate specificity toward UDP-galactose with all three flavonol acceptors (Figs. 4, *A–C* and S3*A*). However, based on the absorbance signal, compared with its favored sugar donor, UDP-glucose, the glycosylation activity was lower when using UDP-galactose. Furthermore, we were able to detect the potency of UGT71G1, UGT78G1, and UGT78K6 toward using UDP-xylose with certain acceptors *in vitro* (Fig. 4, Table 1, and Fig. S3, *B–D*). The latter two enzymes exhibited a considerable activity with this alternative donor substrate at least when using some of the tested flavonol acceptors.

**Table 1:** Detected glycosylation activities of the UGT collection using BiG-HiT assay and LC-MS/MS validation

UGTs Acceptors	UGT enzymes					
	VvGT1	UGT71G1	UGT78G1	UGT78K6	UGT708A6	UGT72B1
Kaempferol	✓ (16)	✓ (15)	✓ (15)	✓ (22)	ND	✓
Kaempferol + UDP-Gal	ND	ND	✓ (15)	✓	ND	ND
Kaempferol + UDP-Xyl	✓	ND	✓	✓	ND	ND
Quercetin + UDP-Glc	✓ (16)	✓ (15)	✓ (15)	✓ (22)	✓	✓
Quercetin + UDP-Gal	ND	ND	✓ (15)	✓	ND	ND
Quercetin + UDP-Xyl	✓ (17)	ND	✓	✓	ND	✓
Myricetin + UDP-Glc	✓ (16)	✓ (15)	✓ (15)	✓ (22)	✓	✓
Myricetin + UDP-Gal	ND	ND	✓	✓	ND	ND
Myricetin + UDP-Xyl	ND	ND	✓	✓	ND	ND
Emodin + UDP-Glc	ND	✓	✓	✓	ND	✓
Emodin + UDP-Gal	ND	ND	ND	ND	ND	ND
Emodin + UDP-Xyl	✓	✓	ND	✓	ND	ND
ε-Rhodomycinone + UDP-Glc	ND	✓	ND	✓	ND	✓
ε-Rhodomycinone + UDP-Gal	ND	ND	ND	ND	ND	ND
ε-Rhodomycinone + UDP-Xyl	ND	✓	ND	ND	ND	ND

Combinations of substrate and enzyme resulting in glycosylation are indicated with check marks (✓) and those that have been previously reported are followed by their reference numbers in parentheses.

Abbreviation: ND not detected

As summarized in Figure 4, *A–C*, all six UGTs were shown to accommodate a minimum of two of the flavonols tested as acceptor substrates at least when UDP-glucose was the sugar donor. These observations were consistent with previous studies on VvGT1, UGT71G1, UGT78G1, and UGT78K6 (15, 16, 22). Excitingly, UGT708A6 and UGT72B1 showed previously unreported *in vitro* substrate tolerance toward at least two tested flavonols, yet the activity was subtle compared with the other UGTs (Fig. 4, *A–C*, Table 1, and Fig. S3, *E* and *F*).

Overall, our screening by absorbance showed several hits for promiscuous GT activity especially among those reactions using flavonoid acceptor substrates as indicated by clear signals more than one standard deviation above the controls. By this criterion, a total of 33 hits were identified (31 with flavonoids and 2 with the anthraquinone emodin). We performed follow-up HPLC-MS/MS not only for the reactions identified as hits but also for those that gave more ambiguous results from screening (29 additional reactions), wherein the average absorbance signals were above those of the controls but had overlapping confidence intervals (±1 standard deviation). From follow-up with HPLC-MS/MS, we identified an additional flavonoid glycosylation reaction (UGT78K6-catalyzed galactosylation of kaempferol [Fig. S3*A*]) and five emodin glycosylation reactions (UGT78G1- and UGT72B1-catalyzed glucosylation, and VvGT1-, UGT71G1-, and UGT78K6-catalyzed xylosylation) (Fig. S3, *G* and *H*) resulting from promiscuous GT activity. Together these results indicate that our initial screen reliably detects glycosylation of flavonoids catalyzed by our panel of UGTs, accurately identifying all but one of the successful reactions as clear hits, but it does not perform as well with anthraquinones. This is probably due not only to the better optimization of the assay toward flavonoids and their glycosides but also to lower activity of the enzymes on anthraquinones in general. We observed that the optical signal from the BiG HiT screen correlates with the quantitation by HPLC-MS (Fig. S4), although for reactions with emodin—on which activity from our panel of UGTs is low—this was near the limit of detection for absorbance readings.

**Figure 5:**
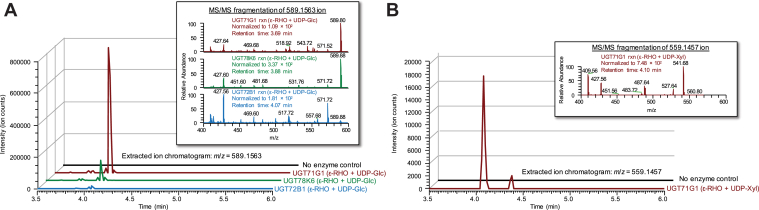
**HPLC-MS analysis of ε-rhodomycinone glycosides.** Extracted ion chromatograms of *A*, glycosylation reactions using ε-rhodomycinone and UDP-glucose as substrates with enzymes UGT71G1 (*red*), UGT78K6 (*green*), UGT72B1 (*blue*), or a no enzyme control (*black*), scanning for ions corresponding to ε-rhodomycinone glucoside ([M - H]-, 589.1563); inset tandem mass spectrometry (MS/MS) fragmentation of 589.1563 ion. *B*, extracted ion chromatograms of glycosylation reactions using ε-rhodomycinone and UDP-xylose as substrates with enzymes UGT71G1 (*red*) or a no enzyme control (*black*), scanning for ions corresponding to ε-rhodomycinone xyloside ([M - H]-, 559.1457); inset MS/MS fragmentation of 559.1457 ion.

While our BiG HiT screen did not reveal hits above background signal using the anthraquinone ε-rhodomycinone as an acceptor substrate, the activity we detected from several UGTs on emodin (another anthraquinone) prompted us to test those same UGTs with ε-rhodomycinone as an acceptor using higher-sensitivity HPLC-MS/MS assays. Intriguingly, glycosylation of ε-rhodomycinone was observed in some of those assays, with four of the seven enzyme and sugar-donor combinations that resulted in emodin glycosylation also leading to ε-rhodomycinone glycosylation (UGT71G1-, UGT78K6-, and UGT72B1-catalyzed glucosylation and UGT71G1-catalyzed xylosylation). The enzymes UGT71G1 and UGT78K6, which performed best in glycosylating emodin (as measured by BiG HiT and HPLC-MS/MS) also performed best on ε-rhodomycinone. Table 1 summarizes previously reported and novel glycosylation activities of our UGT collection. In all, we observed that five of the six UGTs in our collection exhibited novel *in vitro* acceptor specificities at least with one of the anthraquinones (Figs. 4*D* and 5, Table 1, and Fig. S3, *G* and *H*). VvGT1, UGT71G1, UGT78G1, UGT78K6, and UGT72B1 were capable of glycosylating emodin either with UDP-glucose and/or UDP-xylose. Emodin is a plant-derived anthraquinone that has some structural similarities to flavonols (*i.e.*, a tricyclic skeleton and multiple phenolic hydroxyl groups). These structural resemblances may enable the accommodation of emodin in the acceptor pocket of the enzyme. While in general, the enzymes in our panel showed poorer performance on ε-rhodomycinone as a substrate, a major finding of our screen is the identification of three anthraquinone-active UGTs that can generate novel ε-rhodomycinone glycosides (Fig. 5). UGT71G1 catalyzed the transfer of both glucose and xylose to ε-rhodomycinone, whereas UGT78K6 and UGT72B1 catalyzed the transfer of only glucose to ε-rhodomycinone. These activities were verified by LC-MS/MS, wherein distinctive fragmentation profiles were observed by the loss of glucose (162 *m/z*) (Fig. 5*A*) or xylose (132 *m/z*) (Fig. 5*B*) moieties. The ε-rhodomycinone aglycone is of clinical significance as it is the precursor of anticancer anthracyclines including doxorubicin and epirubicin (23). The only structural difference between these two chemotherapeutic molecules is on the sugar residue, which accounts for the reduced cardiotoxicity of epirubicin compared with doxorubicin. Hence, novel ε-rhodomycinone glycosides and enzymes identified in our screen may prove important toward developing glycodiversified anthracycline therapeutics.

## Discussion

High-throughput screening platforms are vital in directed evolution that involve large libraries of mutants and in combinatorial screening to detect novel enzyme activities, substrate specificities, and novel glycosides with biological significance. In this study, we have developed a screening method for assaying natural product GTs in a high-throughput fashion enabled by rapid isolation and detection of chromophoric or fluorescent glycosylated natural products. To the best of our knowledge, the GT assay we developed is the first screening tool designed that uses rapid, nonchromatographic separation of the aglycone and the glycoside product based on polarity, followed by product detection by absorbance/fluorescence. We have demonstrated the compatibility of our plate-based high-throughput screen with both crude lysate and purified GTs, by obtaining Z-factor values greater than 0.5 for the VvGT1 representative enzyme. While the screening effort scales with the library size when performed manually, it is a good strategy for libraries numbering up to thousands of members, as approximately 240 samples can be screened within 2 h. Furthermore, we have designed a device (Fig. S5) to facilitate automation of the liquid–liquid extraction, which consists of discs of silicone-impregnated filter paper placed in a solvent-resistant, 3D-printed housing in a 96-well format. This would enable the solvent extraction of 96 samples simultaneously *via* robotic liquid handling as opposed to the use of an eight-channel pipette, potentially avoiding handling errors.

Using our high-throughput assay, we were able to identify previously unreported donor and acceptor specificities of certain UGTs in our enzyme collection. The preferred sugar donor substrate of many plant UGTs is UDP-glucose, and this is in line with our observations. Yet our results suggest that the substrate specificity of certain UGTs in the collection was not limited to the favored sugar donor, but a few of them could use UDP-galactose and UDP-xylose. However, the difference between the orientation and the number of hydroxyl groups could affect the sugar donor specificity of a particular GT. For example, the orientation of the 4-OH group of the galactose moiety is different from that in UDP-glucose and Wang and colleagues suggested that this opposite positioning could interfere with the enzyme–donor interaction (24). While this was proposed for UGT71G1, we can speculate a similar rationale for UGT78K6 as this enzyme resulted in a reduced preference to UDP-galactose, which was observed by the lower absorbance of the aqueous phase compared with the presence of UDP-glucose.

Many UGTs from our enzyme collection were characterized as flavonoid-glycosyltransferases since these enzymes showed activity with several flavonoids *in vitro* (15, 16, 22). In our analysis, we observed previously unreported flavonol acceptor specificities for UGT708A6 and UGT72B1. *In vitro*, UGT708A6 showed low levels of activity toward quercetin and myricetin, whereas none was observed with kaempferol. However, Casati and colleagues were not able to observe quercetin or kaempferol glycoconjugates when these acceptors were fed exogenously to UGT708A6-expressing *E. coli* cells (25). In a previous *in vitro* study on UGT72B1, Edwards and coworkers did not observe glycosylated flavonol products using quercetin, kaempferol, and myricetin as acceptor substrates (21). Yet, our screen revealed some level of tolerance by this nonflavonoid GT toward the same three flavonols. This discrepancy could be due to several factors that result in higher sensitivity in our BiG HiT assay. Apart from observing novel flavonoid acceptor tolerance, some of the GTs showed substrate flexibility toward anthraquinone acceptors too. Interestingly, our screening tool enabled us to identify three UGTs that were flexible in synthesizing novel anthraquinone glycosides emphasizing the significance of our assay for *in vitro* glycodiversification, given the therapeutic value of anthraquinone drugs. The GTs identified through our strategy and the novel compounds that they produce show promise toward new biocatalytic tools and therapeutics.

Although we have made a considerable improvement in characterizing the substrate flexibility of enzymes in our UGT library, further studies are needed to elucidate the regiochemistry of the produced glycosides, as the HPLC-MS traces reveal distinct peaks for identical masses corresponding to glycosides, which is consistent with different regioisomers produced by the various enzymes (Fig. S3).

In conclusion, we envision that our BiG HiT assay will be an invaluable tool for discovering, analyzing, and engineering GTs in a high-throughput fashion. This can be used to screen any natural product GT that acts on chromogenic/fluorescent nonpolar aglycones including but not limited to flavonoids, anthraquinones, coumarins, and angucyclines with polar sugar donors. Taken together, the simplicity and ease of use without the need for chemically altered substrates or sensors and the compatibility with crude lysates of our BiG HiT screen would make it an attractive screening tool in the field of glycobiology.

## Experimental procedures

### Reagents

Chemicals used in this study were of analytical grade and were commercially purchased. DNase I, RNase A, and lysozyme were purchased from Bio Basic Canada. Restriction endonucleases, T4 DNA ligase, Antarctic phosphatase, and Phusion DNA polymerase were from New England Biolabs. ε-Rhodomycinone was provided by Dr Kristiina Ylihonko.

### Construction of plasmids encoding UGTs

Separate constructs of *UGT71G1*, *UGT78G1*, *UGT78K6*, and *UGT708A6* with *Nde*I and *Xho*I restriction sites were designed in PUC57-Kan vector, and the synthesized constructs were purchased from Bio Basic. Following standard procedures, each gene was excised at *Nde*I and *Xho*I restriction sites, subcloned into pET28a vector, and individual constructs were sequence verified. The *VvGT1* gene had been previously cloned into the pET14b T7 expression vector by Dr Christopher Ford (16) from whom we received the construct as a gift. The *UGT72B1* gene had been previously cloned into the pET30a vector by the group of Dr Gideon Davies at the University of York (26) who kindly provided the construct as a gift.

### Expression and purification of recombinant plant GTs

Each of the UGT constructs in pET vectors were used to transform chemically competent *E. coli* BL21 (DE3) cells and UGT proteins were expressed and purified as described below.

For VvGT1 protein, a previously established protocol was followed (16) with slight modifications. The starter culture was grown overnight at 37 °C, and 1% of it was inoculated into 800 ml of LB broth supplemented with 100 μg/ml of ampicillin. Cells were grown at 16 °C until *A*_600_ was between 0.4 to 0.6 and protein expression was induced with 0.4 mM IPTG. The cultures were further incubated for an additional 24 h at 16 °C and the cells were harvested by centrifugation (10,000*g*, 30 min, 4 °C). The cell pellet was resuspended in 40 ml of lysis buffer (20 mM Tris pH 8, 100 mM NaCl, 10 mM 2-mercaptoethanol, 10% v/v glycerol). DNase I, RNase A, and lysozyme (5 μg/ml of each) and one mini tablet of protease inhibitor cocktail (EDTA-free) were added. Following cell disruption by sonication with a 5 s pulse on, 15 s pulse off for a total pulse time of 3 min at 25% amplitude (Fisherbrand Model 505 Sonic Dismembrator), the insoluble components were pelleted by centrifugation at 15,000*g* for 30 min at 4 °C. A 1-ml Ni-NTA resin column (MCLAB) was pre-equilibrated with 10 ml of wash buffer (20 mM Tris pH 8, 100 mM NaCl, 10 mM 2-mercaptoethanol, 10% v/v glycerol, 10 mM imidazole), and the filtered supernatant (0.22-μm filter, UltiDent) was loaded to the column. Subsequently, the column was washed with 10 column volumes of wash buffer and the expressed VvGT1 protein was isocratic eluted at 4 °C by gravity through 8 column volumes of elution buffer (20 mM Tris pH 8, 100 mM NaCl, 10 mM 2-mercaptoethanol, 10% v/v glycerol, and 300 mM imidazole). The fractions containing VvGT1 protein were pooled, and buffer exchanged using 10DG desalting column (Bio-Rad) with 4 ml of storage buffer (50 mM Tris pH 7.5, 150 mM NaCl, 10 mM 2-mercaptoethanol, and 10% v/v glycerol), and aliquots of protein were stored at −80 °C.

For the remaining five UGTs, 1% of starter cultures that had grown overnight at 37 °C were inoculated into separate 800 ml volumes of LB broths containing 50 μg/ml of kanamycin. The cultures were incubated at 37 °C until *A*_600_ of 0.4 to 0.8 was reached and induced with 0.5 mM IPTG. All the cultures were overnight grown at 16 °C, and bacterial cells were harvested by centrifugation at 10,000*g* for 30 min at 4 °C. Cell lysis, protein purification, buffer exchange, and storage were performed as explained above using the following buffers: lysis/wash buffer: 50 mM Tris pH 8, 500 mM NaCl, 10 mM 2-mercaptoethanol, and 10 mM imidazole; elution buffer: 50 mM Tris pH 8, 500 mM NaCl, 10 mM 2-mercaptoethanol, and 250 mM imidazole; and storage buffer: 50 mM Tris pH 7.5, 150 mM NaCl, and 5% v/v glycerol.

### Biphasic glycosyltransferase assay validation using purified VvGT1 enzyme

The BiG HiT assay validation was carried out in a 96-well PCR plate (VWR) with eight replicates. A volume of 10 μl of purified VvGT1 protein (0.25 mg/ml in total reaction volume) was added to a 20-μl substrate mixture containing 10 mM Tris pH 7.8, 1.5 mM quercetin (3% dimethyl sulfoxide), and 3 mM UDP-glucose, and the total reaction volume was 30 μl. As the no enzyme control, 10 μl of assay control buffer (50 mM Tris pH 7.5, 150 mM NaCl) was added to the substrate mixture. The reactions were incubated at 30 °C for 2 h and terminated by incubating at 90 °C for 3 min. The insoluble components were pelleted out by centrifugation at 3000*g* for 5 min. A volume of 25 μl of the supernatant was transferred to a new well and one volume of ethyl acetate:chloroform (3:1 v/v) was added as the organic phase. The content was mixed using an eight-channel pipettor (Corning Lambda Plus), and liquid–liquid extraction was performed on a Whatman 1PS phase separator filter paper (GE Healthcare). The solvent extraction step was repeated, and 15 μl of the aqueous phase was transferred to wells of 96-well half area plate (Corning). The sample was diluted with 1 volume of distilled water and 2 volumes of 0.2% 2-aminoethyl diphenylborinate fluorescent probe in 20% ethanol. The absorbance at 394 nm was measured using a CLARIOstar monochromator microplate reader (BMG Labtech), and the data were received from MARS data analysis software 3.20 R2.

### Biphasic glycosyltransferase assay validation using crude VvGT1 enzyme

To assay the compatibility of crude lysate with the developed high-throughput screen, VvGT1 protein lysate was prepared as follows: pET14b plasmid encoding VvGT1 was transferred into chemically competent XJb (DE3) autolysis cells. The cells were grown in 1 ml of LB broth containing 100 μg/ml of ampicillin at 37 °C for 24 h, and as the control XJb (DE3) cells harboring an empty pET14b vector was used. A volume of 7.5 μl of inoculum was added into V-bottom 96-well microplates (NUNC) having 150 μl LBE-5052 autoinduction medium supplemented with 100 μg/ml of ampicillin and 3 mM arabinose (eight replicates). The cells were grown at 30 °C for an additional 24 h. The cells were pelleted by centrifuging at 3000*g* for 10 min, and the cell pellets were resuspended in 30 μl of 10 mM Tris pH 7.8. The cells were frozen at −80 °C for 1 h, then thawed at 30 °C. Following the addition of 10 μl of a mixture containing 20 μg/ml DNase I, 20 μg/ml RNase A, and 20 μg/ml lysozyme in 10 mM Tris buffer, pH 7.8, the cell suspension was incubated at room temperature for 15 min. Cell debris were removed by centrifugation (3000*g* for 10 min at 4 °C) and 10 μl of crude lysate was used for the above explained high-throughput assay validation procedure.

### *In vitro* enzymatic synthesis and purification of UDP-xylose

UDP-xylose was enzymatically prepared from UDP-glucose in a one-pot two-step reaction following a previously described procedure (27).

### High-throughput combinatorial screening of promiscuous plant UGT collection

The purified UGT enzyme collection (along with VvGT1) and a panel of substrates were combinatorially screened using the high-throughput assay we developed. Herein, quercetin, kaempferol, myricetin, emodin, and ε-rhodomycinone were used as acceptors and UDP-glucose, UDP-galactose, and UDP-xylose were used as sugar donors. For each acceptor substrate, a 96-well PCR plate (VWR) was used with different combinations of UGTs and sugar donors, and the screening was performed as explained above with a minimum of two replicates. Two negative controls, either without sugar donors or without UGTs were performed. The organic solvent mix for flavonoids and anthraquinones were ethyl acetate:chloroform (3:1 v/v) and ethyl acetate:chloroform (1:3 v/v), respectively. For flavonoid acceptors, 0.2% of 2-aminoethyl diphenylborinate was used.

### Validation of positive hits by HPLC-MS/MS analysis

The positive reactions of the combinatorial high-throughput screen were analyzed by performing HPLC-MS/MS analysis using an Acquity BEH C18 column (2.1 × 50 mm, 1.7 μm) connected to an Agilent 1260 Infinity II UHPLC system (Agilent Technologies) equipped with a Thermo-Finnigan 7 T LTQ-FT mass spectrometer. A volume of 5 μl of the diluted sample (1:1 using distilled water) was injected, and chromatographic separation was performed at a flow rate of 0.3 ml/min with a gradient of 5% to 80% buffer B over 5 min, followed by two isocratic steps with 90% buffer B over 0.5 min, and 5% buffer B over 2.5 min where buffer A was water with 0.1% formic acid and buffer B was acetonitrile with 0.1% formic acid. The analytes were detected using mass spectrometry in negative mode, and MS/MS fragmentation was done for selected *m/z* ratios at an isolation window of 2.5 Da, activation time of 30 s, and using 15 and 20 normalized collision energies (cid).

## Data availability

All data presented are contained within the article and supporting information.

## Supporting information

This article contains supporting information.
