# Supplementary material for: A “biphasic glycosyltransferase high-throughput screen” identifies novel anthraquinone glycosides in the diversification of phenolic natural products
Source: J Biol Chem. 2023 Jan 20;299(3):102931. doi: 10.1016/j.jbc.2023.102931 (PMC9950524; doi:10.1016/j.jbc.2023.102931)
Supplement: Supplemental Figures S1–S5 [file mmc1.pdf]

Supporting Information for

A "biphasic glycosyltransferase high-throughput screen" identifies novel anthraquinone glycosides in the diversification of phenolic natural products

F. Ifthiha Mohideen[1,†] and David H. Kwan[1,2,3,*]

[1] Department of Biology, Centre for Applied Synthetic Biology, and Centre for Structural and Functional Genomics, Concordia University, 7141 Sherbrooke Street West, Montreal, Quebec, Canada H4B 1R6

[2] Department of Chemistry and Biochemistry, Concordia University, 7141 Sherbrooke Street West, Montreal, Quebec, Canada H4B 1R6

[3] PROTEO, Quebec Network for Research on Protein Function, Structure, and Engineering, Quebec City, Quebec, Canada G1V 0A6

[†] Current address: Department of Chemistry, University of Alberta, 11227 Saskatchewan Drive, Edmonton, AB, Canada T6G 2G2

[*]Corresponding author
Tel: (514) 848-2424 x7329
Email: david.kwan@concordia.ca

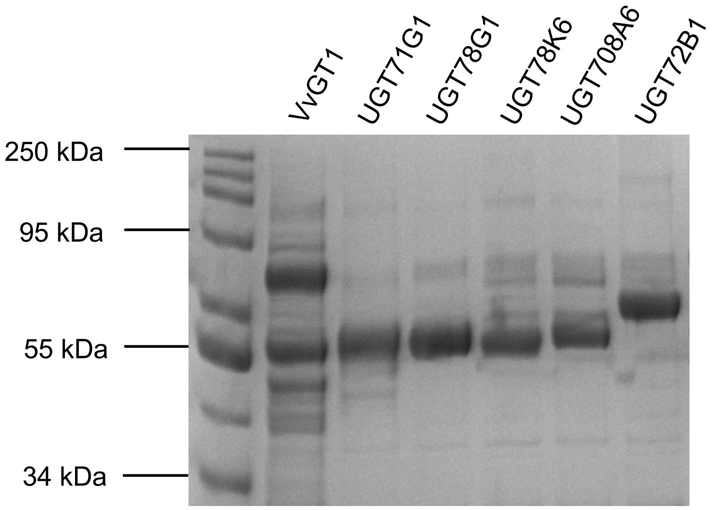

Fig. S1: Bolt^{TM} 4-12% Bis-Tris gel of purified UGT library. The proteins were overexpressed in *E. coli* strains and Ni-NTA purified. Following SDS-PAGE (0.25 – 0.50 mg/mL of final protein concentrations were loaded), the proteins were visualized by staining with Coomassie blue. The molecular weights of the proteins were as follows: VvGT1: 50 kDa, UGT71G1: 52 kDa, UGT78G1: 50 kDa, UGT78K6: 49 kDa, UGT708A6: 51 kDa, and UGT72B1: 53 kDa.

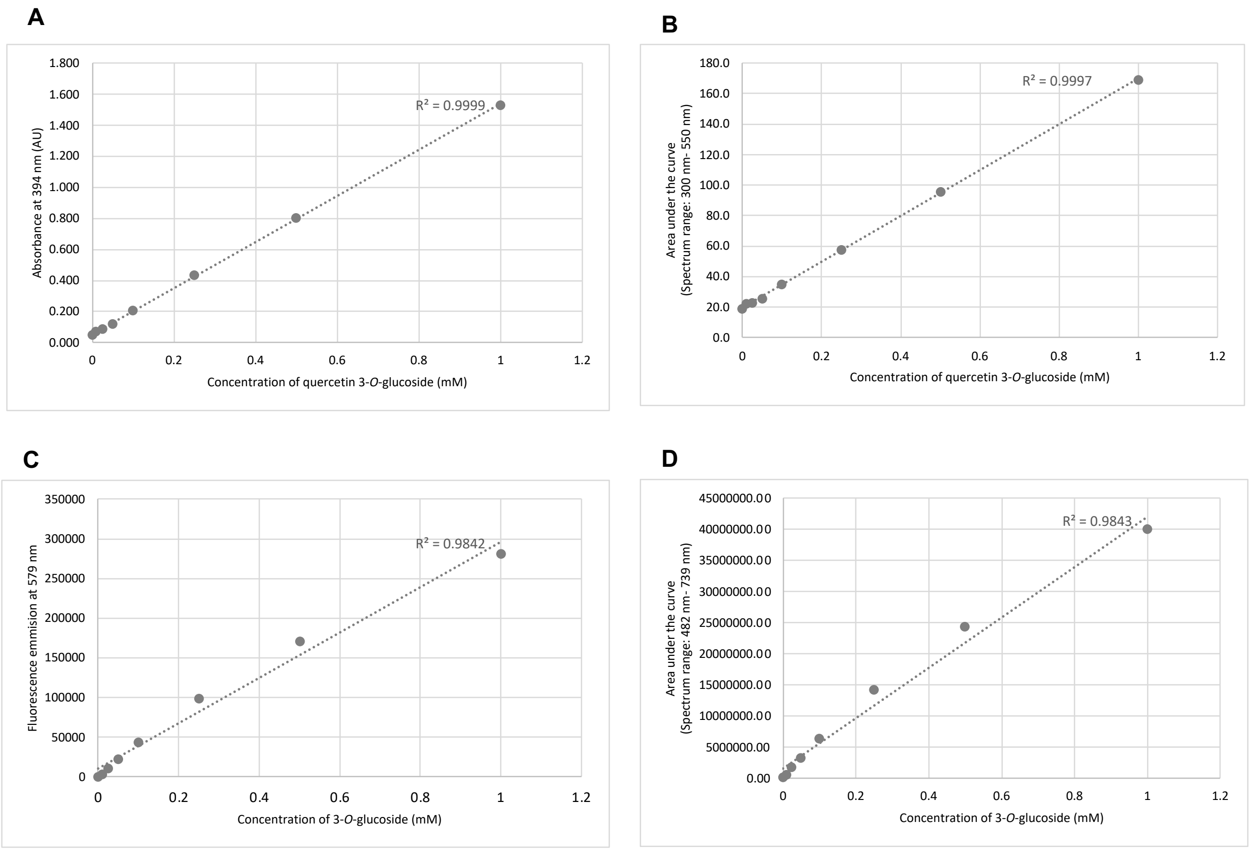

Fig. S2: Standard curves obtained for quercetin 3-*O*-glucoside. A. Absorbance at 394 nm vs concentration, B. Area under the curve for absorbance vs concentration, C. Fluorescence emission at 579 nm vs concentration, and D. Area under the curve for fluorescence vs concentration.

Standard curve generation for quercetin 3-*O*-β-glucoside

8 different concentrations of quercetin 3-*O*-β-glucoside (0 mM, 0.01 mM, 0.025 mM, 0.05 mM, 0.1 mM, 0.25 mM, 0.5 mM, and 1 mM) were made in the assay buffer containing 25 mM Tris pH 7.5, 50 mM NaCl, and 2% DMSO. Each concentration was done in triplicates for either absorbance or fluorescence. 15 µL was transferred to wells of 96-well clear half area plate (Corning). The sample was diluted with 1 volume of distilled water and 2 volumes of 0.2 % 2-aminoethyl diphenylborinate fluorescent probe in 20 % ethanol. Wells were mixed and the absorbance values at varying wavelengths were measured using a CLARIOstar monochromator microplate reader (BMG Labtech). For fluorescence at 454 nm excitation, emission at varying wavelengths was measured using 96-well black half area plate (Costar).

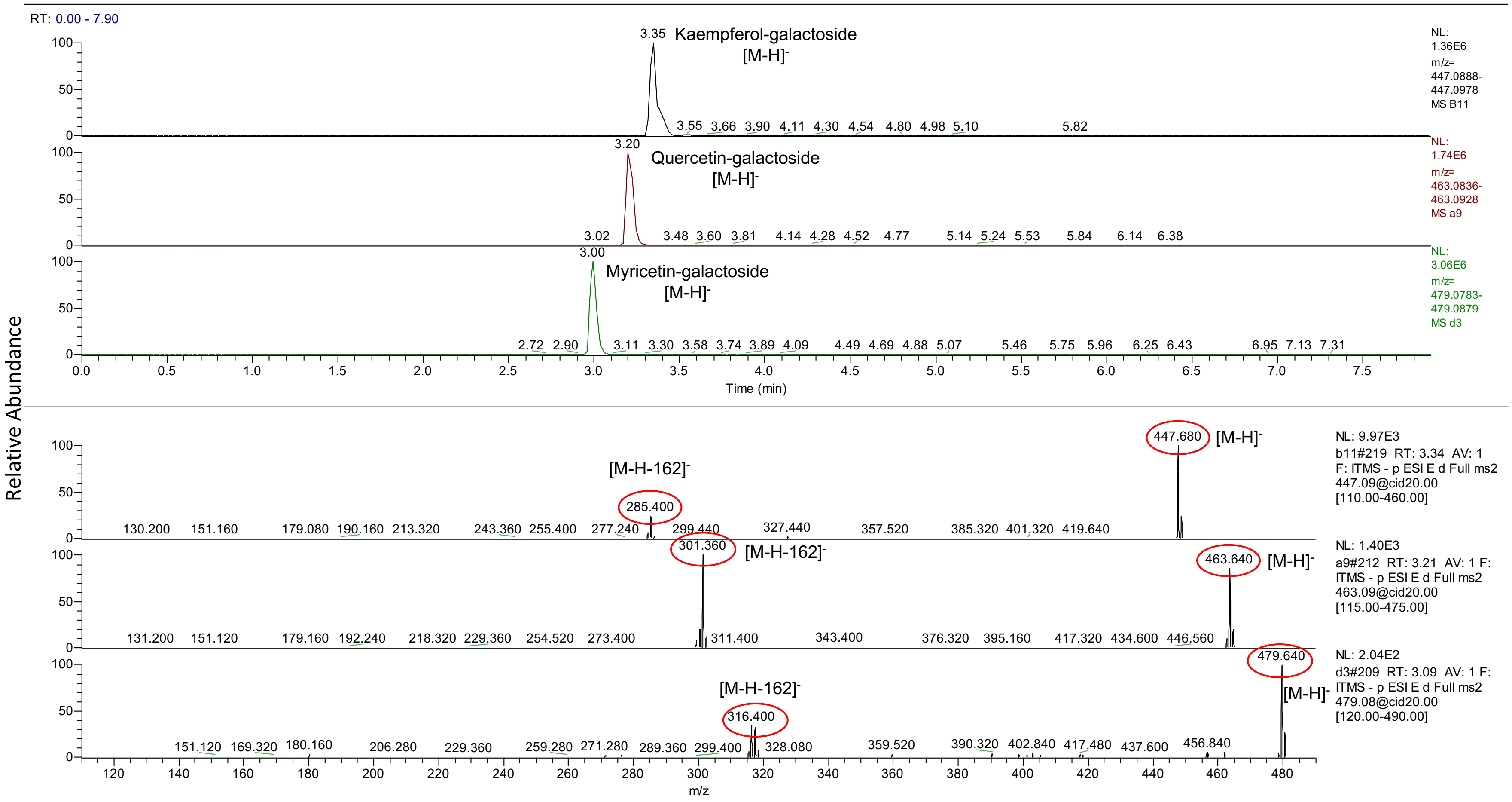

Fig. S3A: LC-MS/MS analysis of UGT78K6 activity with kaempferol, quercetin, and myricetin acceptors in the presence of UDP-galactose. Top 3 panels: LC chromatograms. Bottom 3 panels: respective MS/MS fragmentation profiles at 20 cid for molecules eluted at the peak retention times (RT) indicated. Sample were analyzed in negative mode and loss of galactose is indicated by -162.

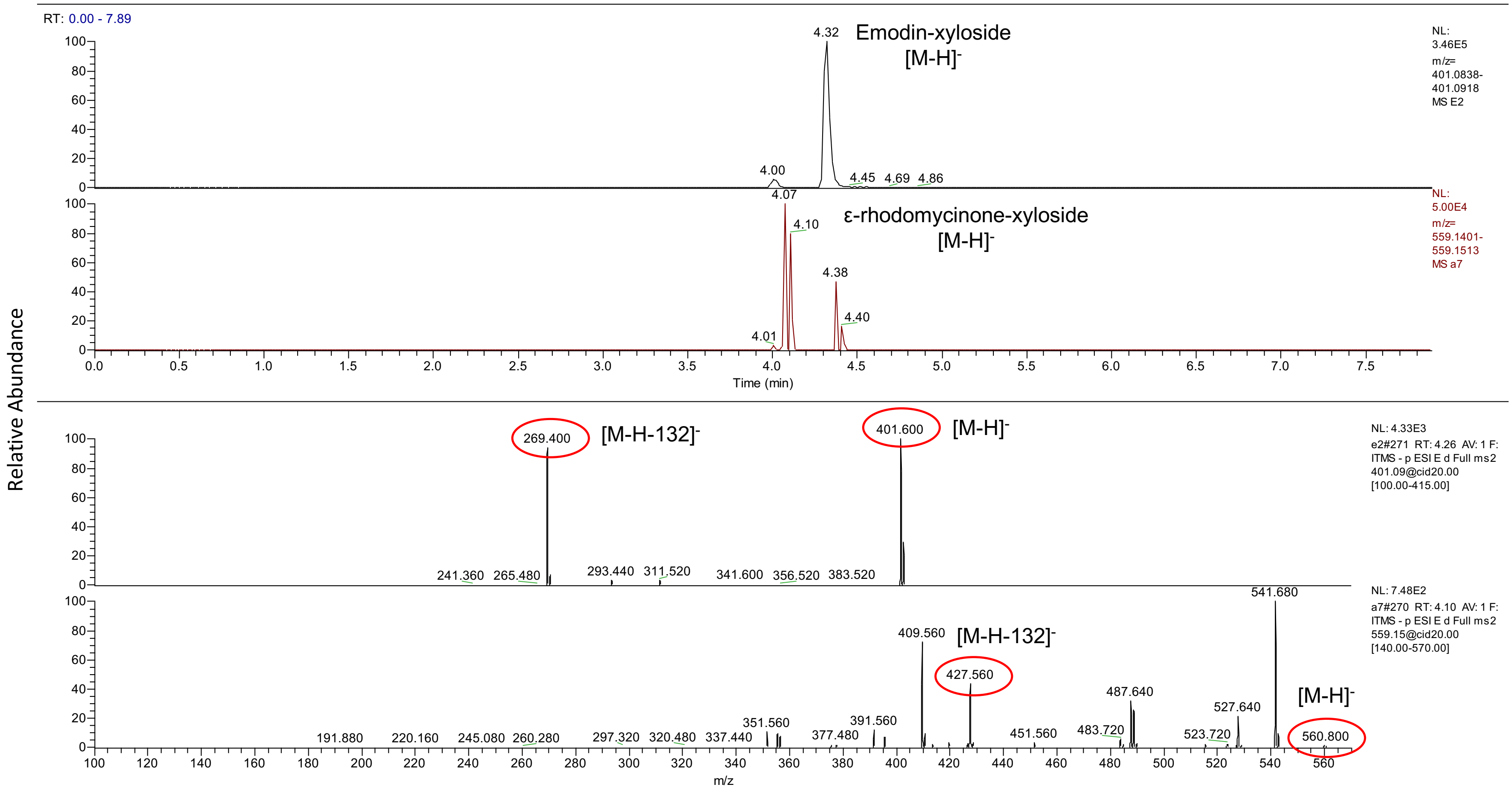

Fig. S3B: LC-MS/MS analysis of UGT71G1 activity with emodin and ε-rhodomycinone acceptors in the presence of UDP-xylose. Top 2 panels: LC chromatograms. Bottom 2 panels: respective MS/MS fragmentation profiles at 20 cid for molecules eluted at the peak retention times (RT) indicated. Sample were analyzed in negative mode and loss of xylose is indicated by -132.

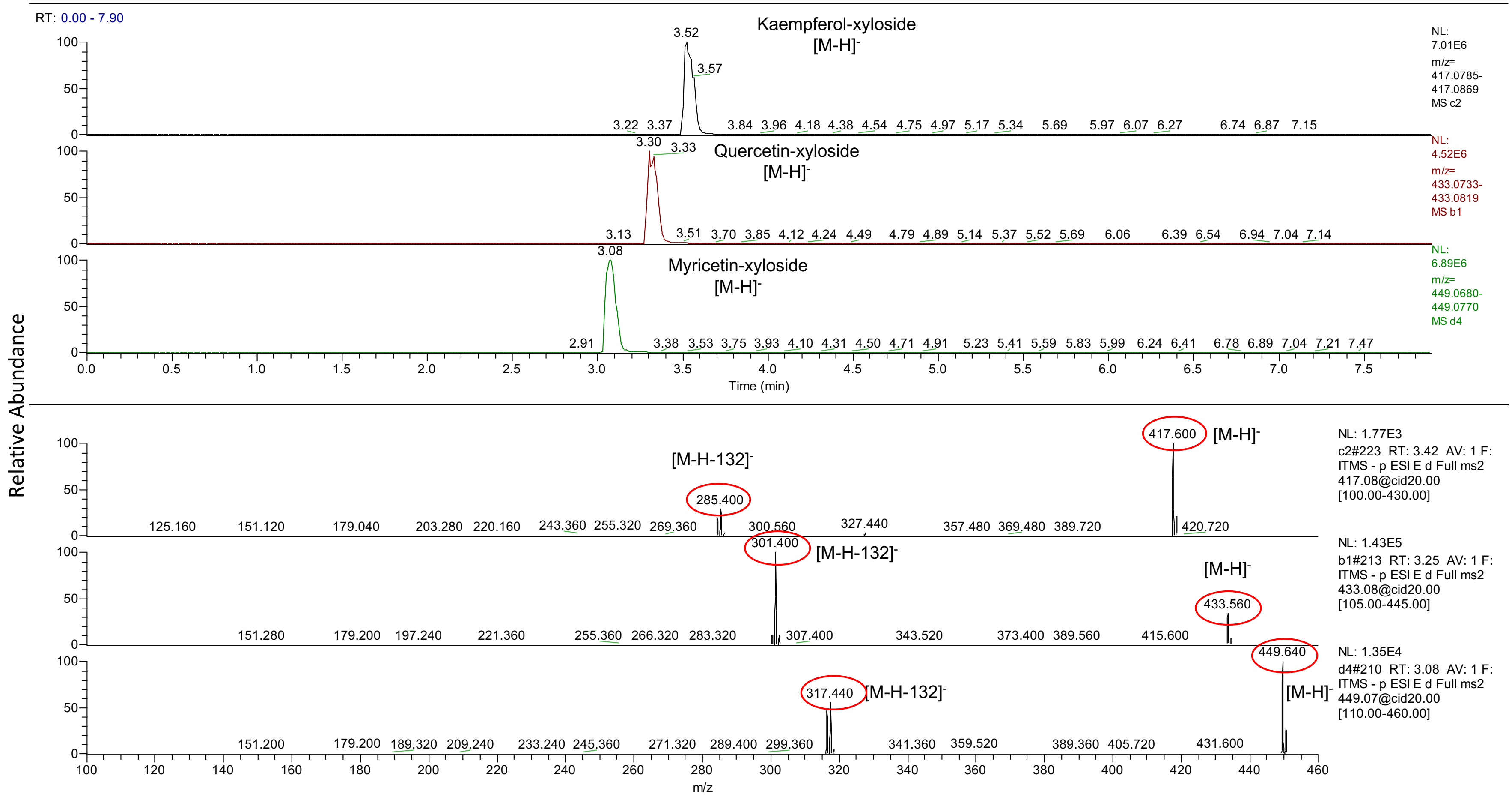

Fig. S3C: LC-MS/MS analysis of UGT78G1 activity with kaempferol, quercetin, and myricetin acceptors in the presence of UDP-xylose. Top 3 panels: LC chromatograms. Bottom 3 panels: respective MS/MS fragmentation profiles at 20 cid for molecules eluted at the peak retention times (RT) indicated. Sample were analyzed in negative mode and loss of xylose is indicated by -132.

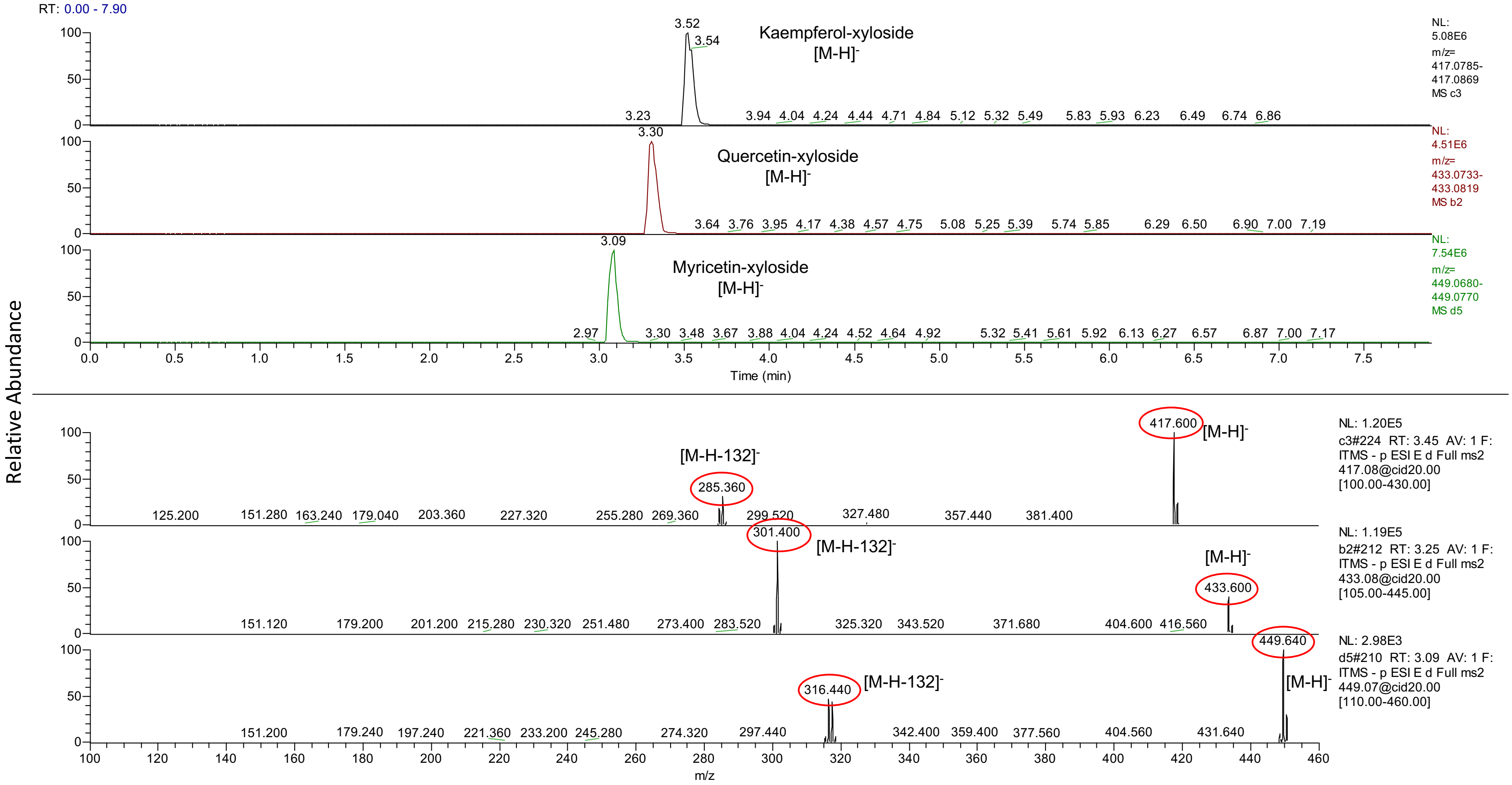

Fig. S3D: LC-MS/MS analysis of UGT78K6 activity with kaempferol, quercetin, and myricetin acceptors in the presence of UDP-xylose. Top 3 panels: LC chromatograms. Bottom 3 panels: respective MS/MS fragmentation profiles at 20 cid for molecules eluted at the peak retention times (RT) indicated. Sample were analyzed in negative mode and loss of xylose is indicated by -132.

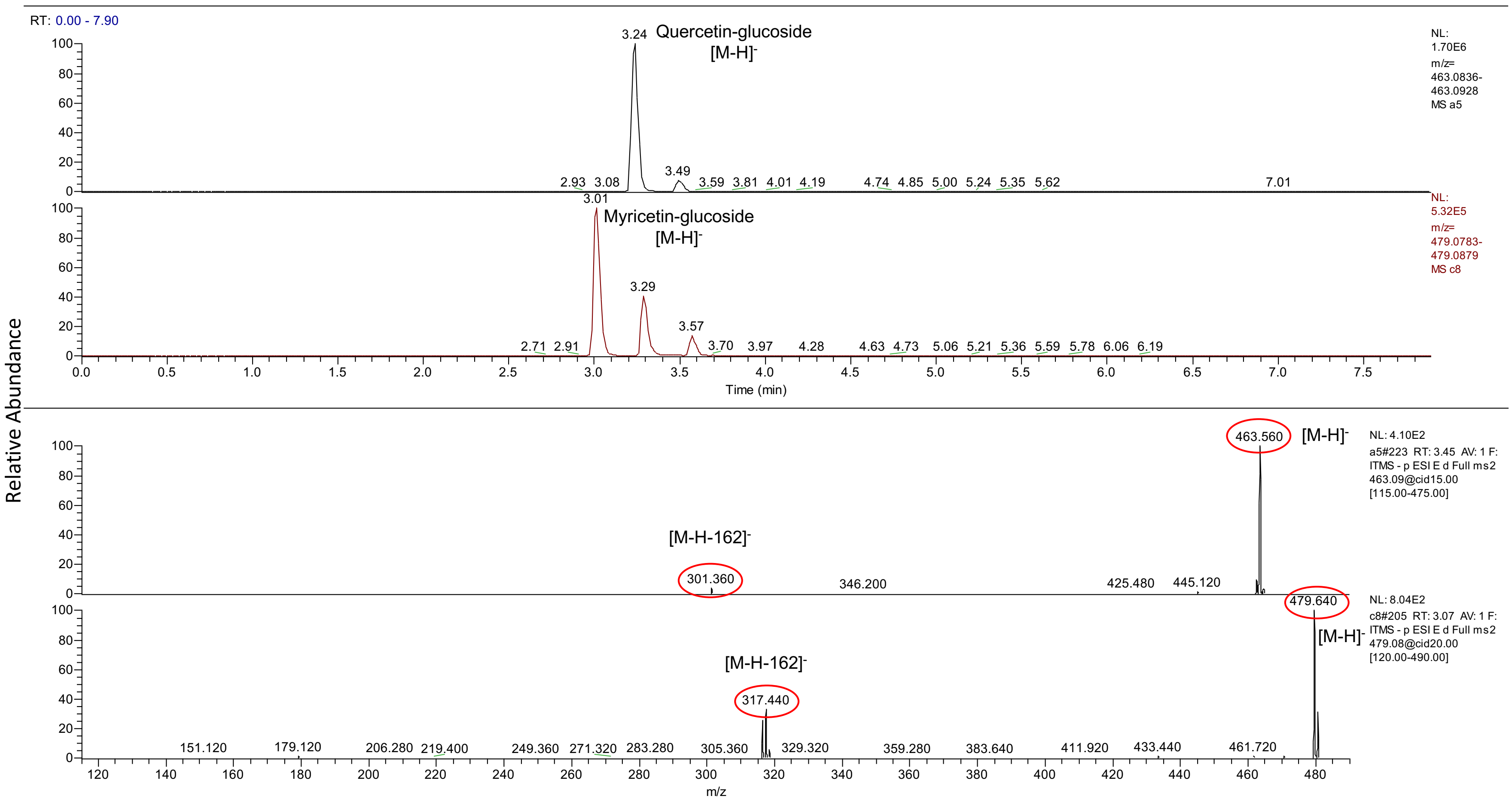

Fig. S3E: LC-MS/MS analysis of UGT708A6 activity with quercetin and myricetin acceptors in the presence of UDP-glucose. Top 2 panels: LC chromatograms. Bottom 2 panels: respective MS/MS fragmentation profiles at 15 and 20 cid for molecules eluted at the peak retention times (RT) indicated. Sample were analyzed in negative mode and loss of glucose is indicated by -162.

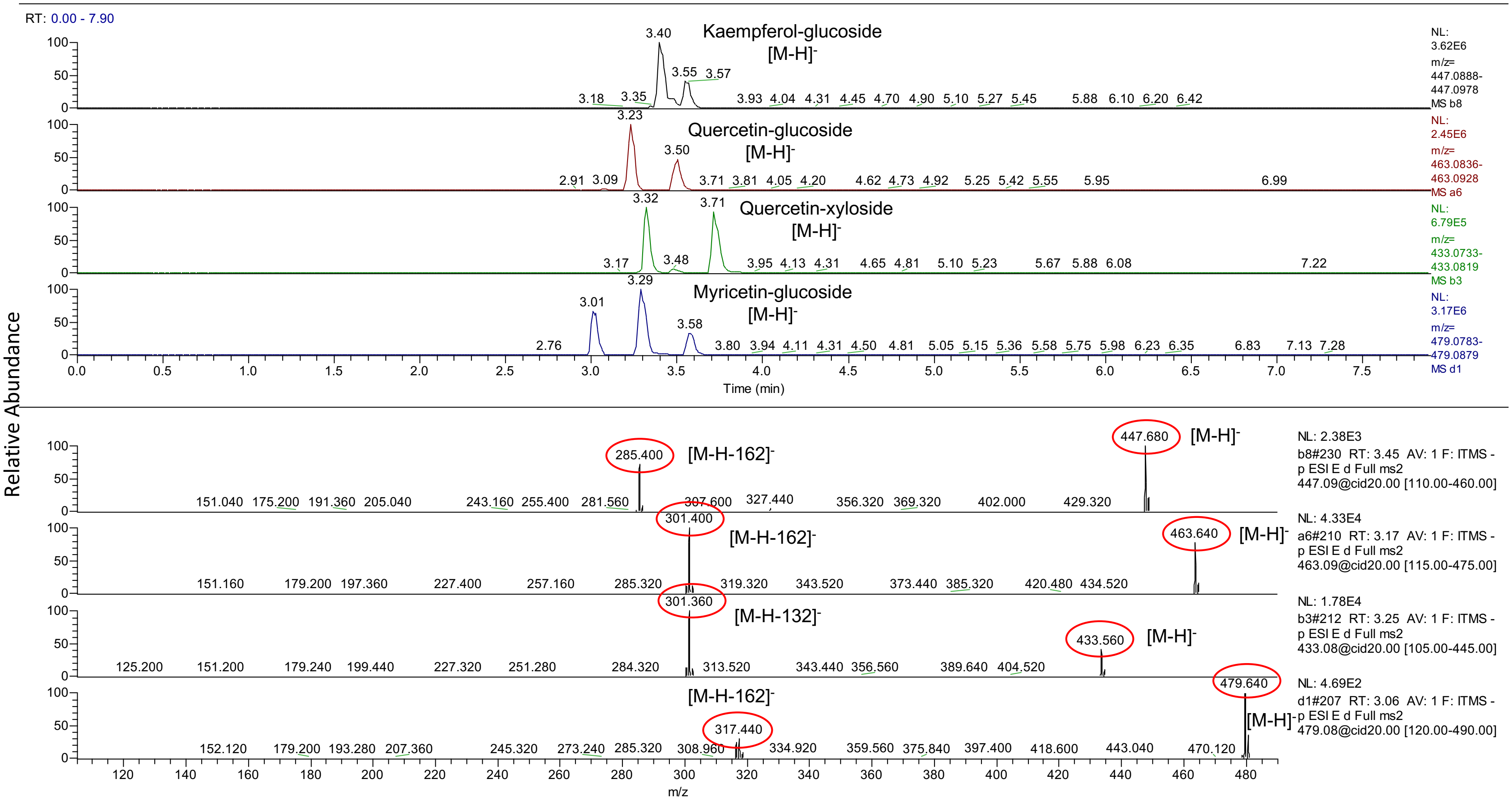

Fig. S3F: LC-MS/MS analysis of UGT72B1 activity with kaempferol, quercetin, and myricetin acceptors in the presence of UDP-glucose/ UDP-xylose. Top 4 panels: LC chromatograms. Bottom 4 panels: respective MS/MS fragmentation profiles at 20 cid for molecules eluted at the peak retention times (RT) indicated. Sample were analyzed in negative mode and loss of glucose and xylose are indicated by -162 and -132, respectively.

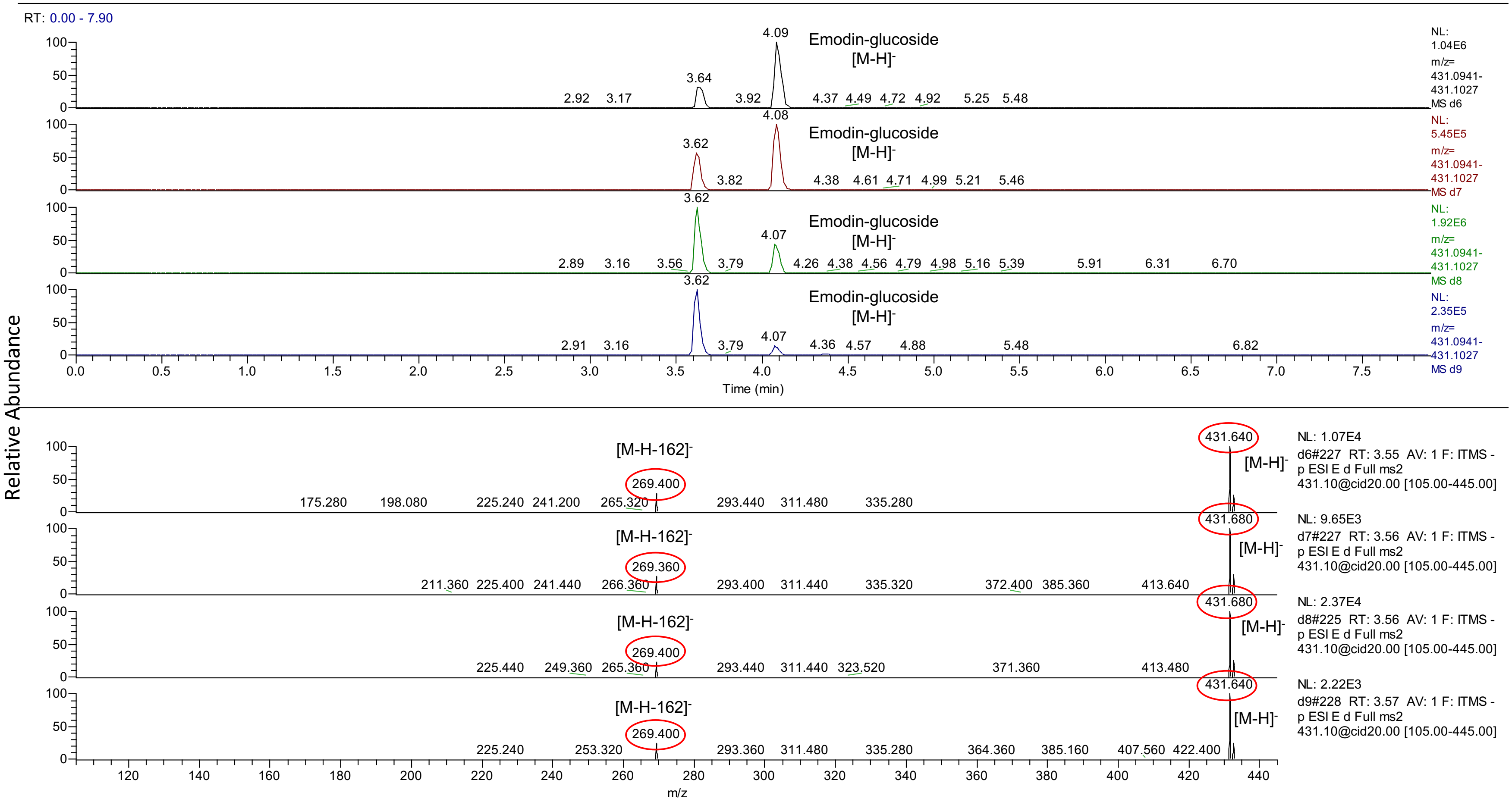

Fig. S3G: LC-MS/MS analysis of UGT activity with emodin acceptor in the presence of UDP-glucose. Top 4 panels: LC chromatograms of UGT71G1, UGT78G1, UGT78K6, and UGT72B1 activities (from top to bottom). Bottom 4 panels: respective MS/MS fragmentation profiles at 20 cid for molecules eluted at the peak retention times (RT) indicated. Sample were analyzed in negative mode and loss of glucose is indicated by -162.

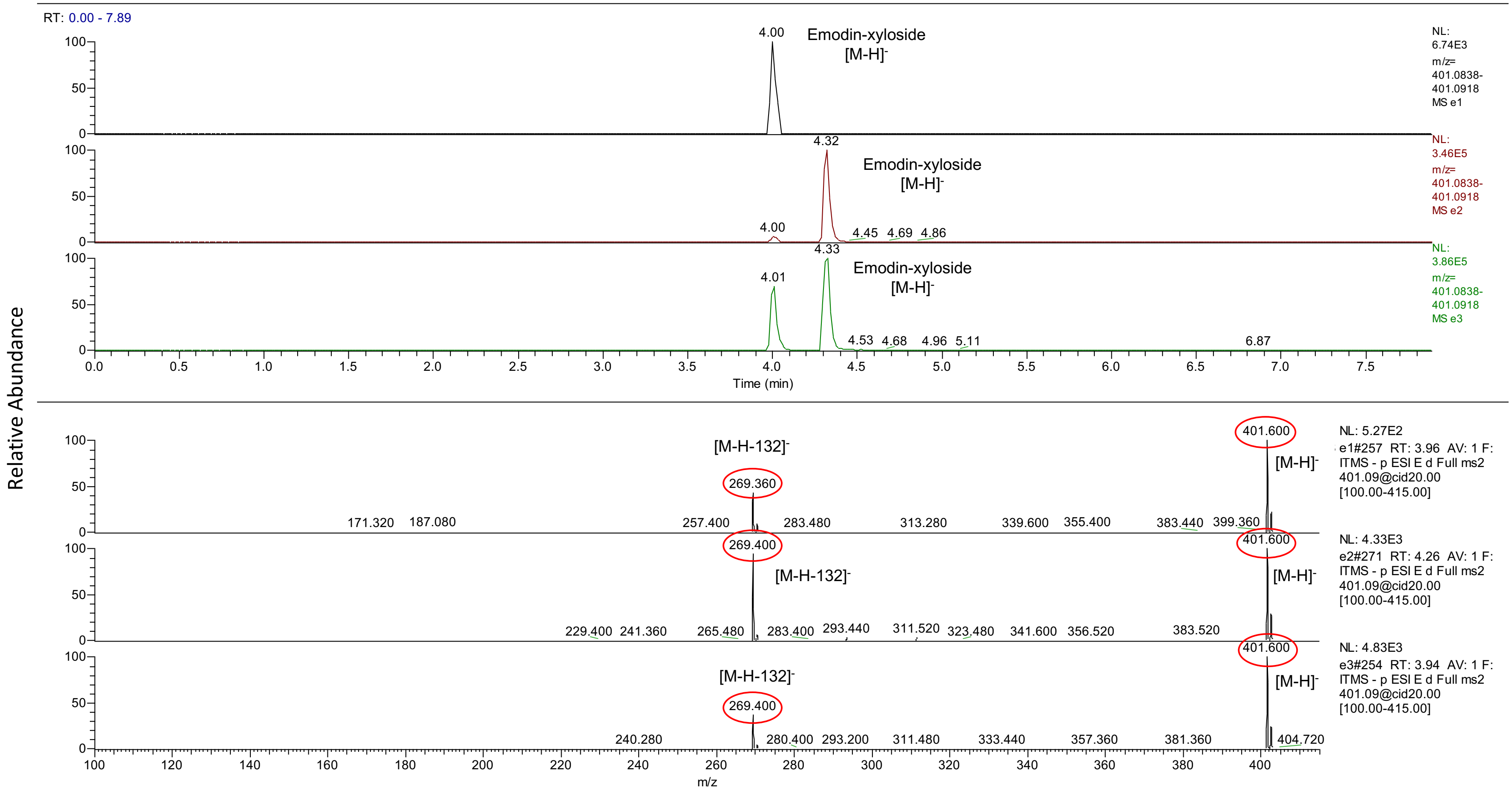

Fig. S3H: LC-MS/MS analysis of UGT activity with emodin acceptor in the presence of UDP-xylose. Top 3 panels: LC chromatograms of VvGT1, UGT71G1, and UGT78K6 activities (from top to bottom). Bottom 3 panels: respective MS/MS fragmentation profiles at 20 cid for molecules eluted at the peak retention times (RT) indicated. Sample were analyzed in negative mode and loss of xylose is indicated by -132.

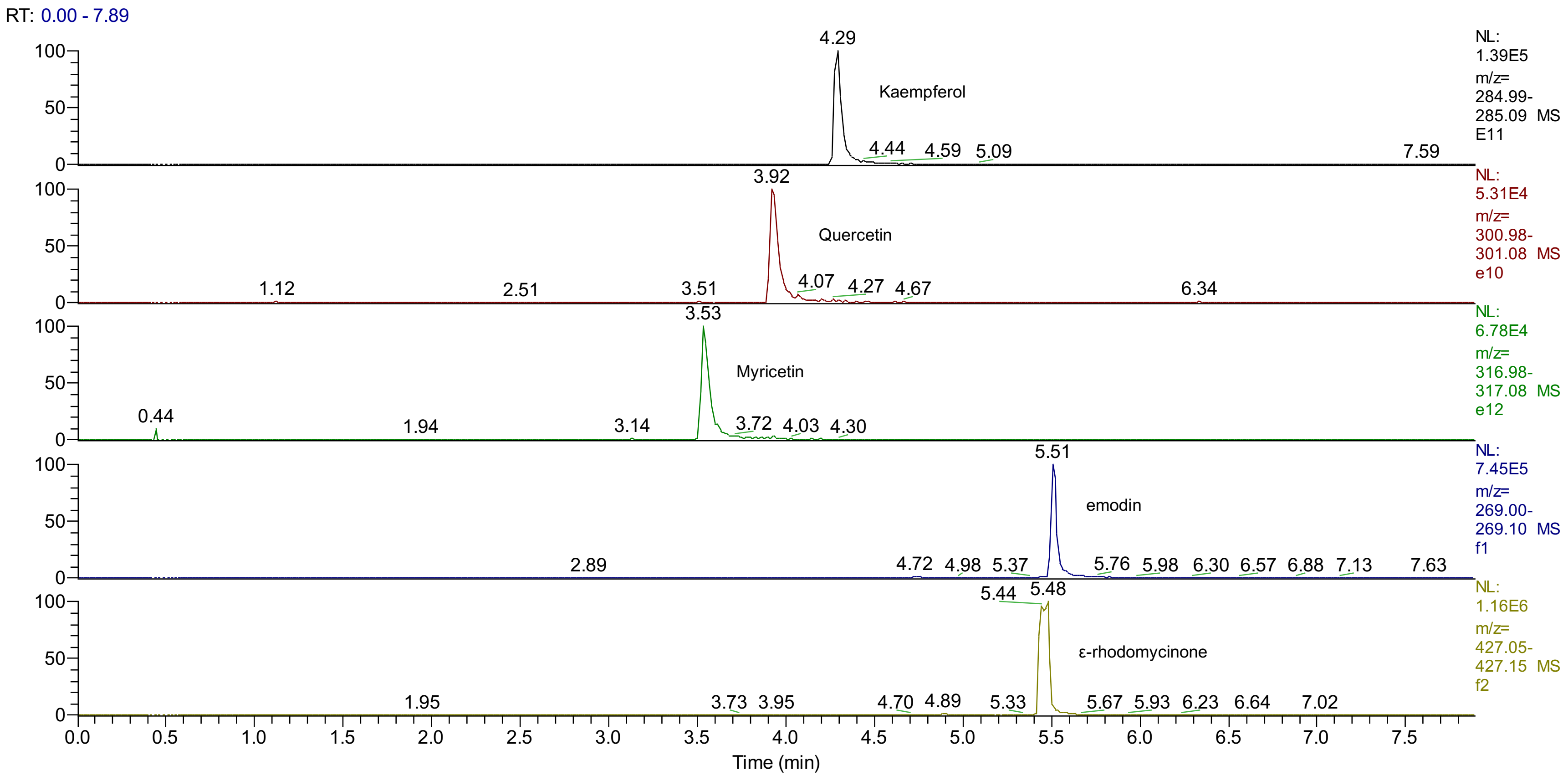

Fig. S3I: LC-MS traces (extracted ion chromatograms) of unmodified acceptor aglycones: kaempherol, quercetin, myricetin, emodin, and ε-rhodomycinone

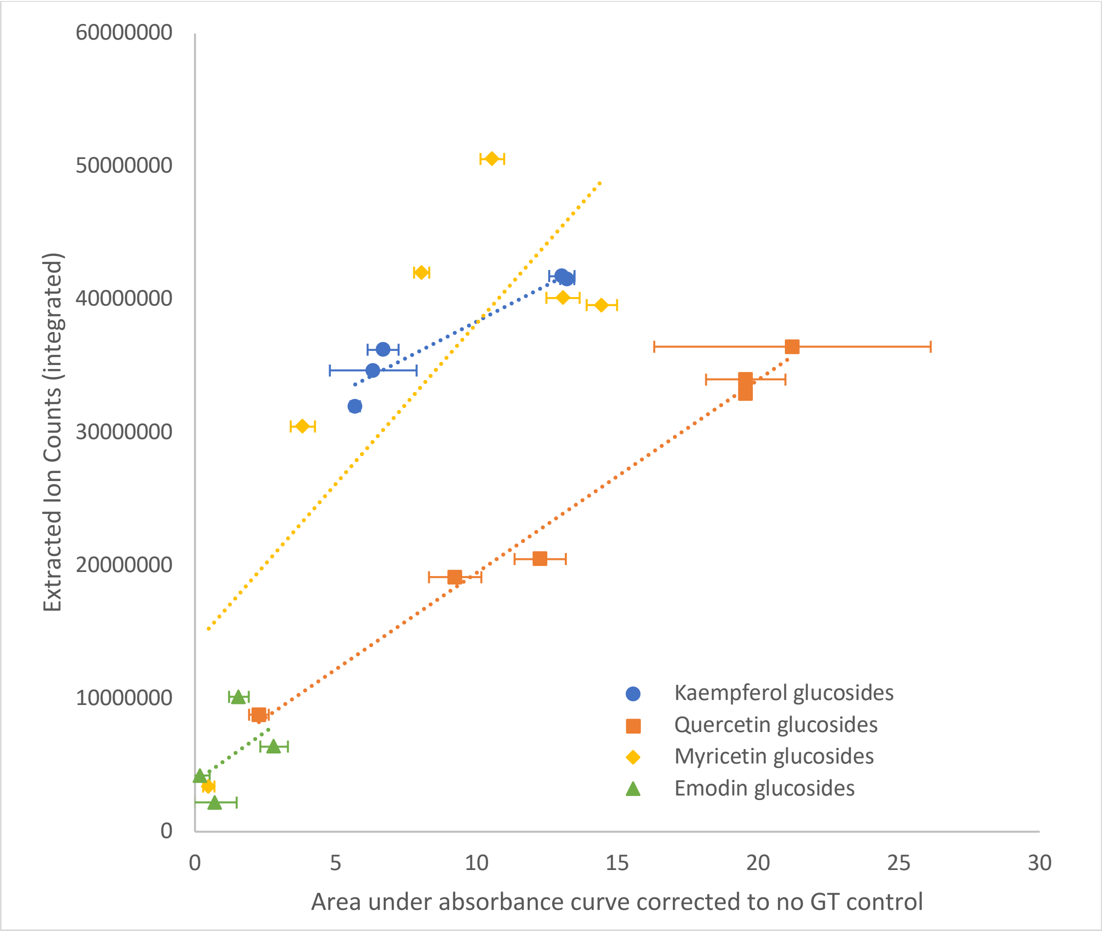

Fig. S4: Plot of absorbance signals from BiG HiT assays of glucosylation reactions (UDP-Glc as donor substrate) vs. signal from HPLC-MS (extracted ion counts of $[M – H]^-$, $[M + Cl]^-$, and $[M + CH_3COO]^-$ showing correlation between signals.

A

B

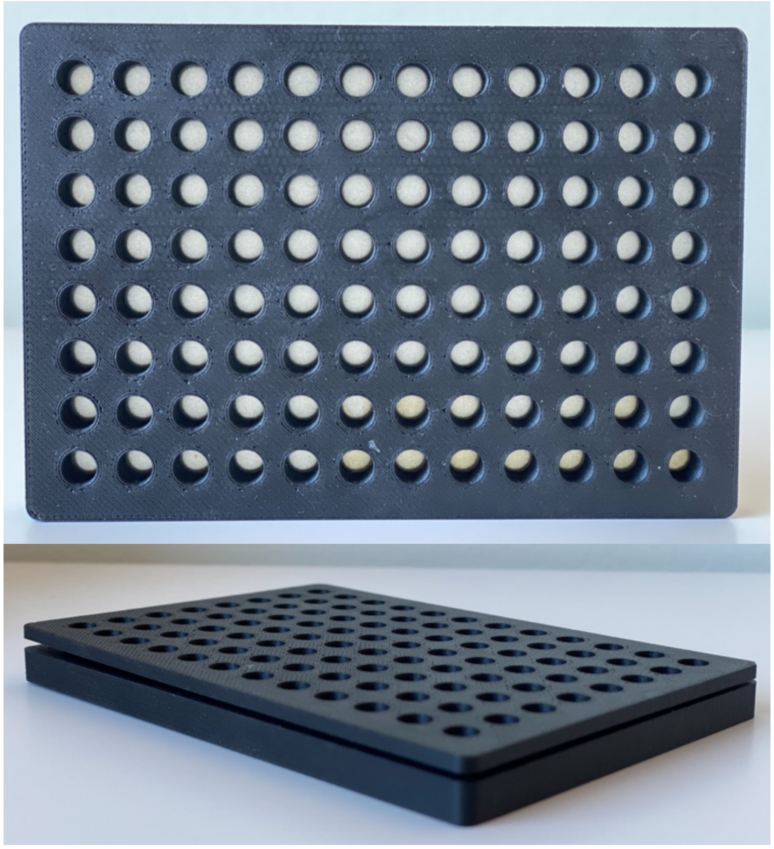

Fig. S5: A, the top and B, side view of the device designed to facilitate the automation of liquid-liquid extraction step in BiG HiT assay. Discs of silicone-impregnated filter paper are sandwiched between the two 3D printed housings.
